# DART-VAG: initial clinical experience with a novel autologous technique for penile girth augmentation

**DOI:** 10.1007/s00345-026-06474-2

**Published:** 2026-05-31

**Authors:** Ubirajara Barroso Jr., Isabela Alvim Barroso, Geraldo Ramos Ribeiro Filho, Lucas Teixeira E. Aguiar Batista, Marco Aurélio Silva Lipay

**Affiliations:** 1 Division of Urology, Federal University of Bahia, Av Alphaville, 335, Rua Pajuçara, Alphaville 1, Salvador, Bahia 41701015 Brazil; 2 São Luiz Jabaquara Hospital, São Paulo, Brazil; 3 Bahiana School of Medicine and Public Health, Salvador, Brazil

**Keywords:** Penile girth augmentation, Surgical flaps, Tunica vaginalis, Patient reported outcome measures, Body image

## Abstract

**Purpose:**

Dissatisfaction with penile girth is an increasingly recognized concern among men and can cause psychosocial distress despite normal anatomy. Existing augmentation techniques are often associated with limited durability or procedure-related morbidity. This study presents the initial clinical experience with a novel autologous penile girth augmentation technique that combines dartos fascia and tunica vaginalis flap (DART-VAG).

**Methods:**

This was a prospective, non-randomized interventional study evaluating the DART-VAG technique. Penile length and circumference were measured preoperatively and at a 6-month follow-up. Psychosexual outcomes were assessed in a subset of patients using the Degree of Distress due to size concerns (DDC) questionnaire and the Male Genital Self-Image Scale (MGSIS). Postoperative complications and patient satisfaction were prospectively recorded.

**Results:**

Thirty-two adult men underwent penile girth augmentation with DART-VAG between November 2021 and August 2025. No intraoperative complications occurred. Mean penile length increased from 6.2 ± 1.67 cm to 8.7 ± 1.46 cm (*p* < 0.001), and mean circumference increased from 9.4 ± 1.08 cm to 11.2 ± 1.15 cm (*p* < 0.001). No patient reported loss of achieved dimensions, and all patients (100%) reported satisfaction at 6 months. Wound dehiscence occurred in four patients (12.5%), most of whom were managed conservatively; one patient (3.1%) required surgical drainage of a scrotal hematoma. Total DDC scores decreased significantly after surgery, and MGSIS improved in specific domains.

**Conclusions:**

DART-VAG is a feasible technique for penile girth augmentation, providing significant gains, acceptable complications, and favorable psychosexual outcomes. Larger studies with longer follow-up are needed to confirm our findings.

**Supplementary Information:**

The online version contains supplementary material available at 10.1007/s00345-026-06474-2.

## Introduction

 Penile size is a well-recognized concern among men and has been shown to affect male self-perception, sexual confidence, and overall psychosocial well-being [1–3]. In this context, dissatisfaction regarding length and girth has led an increasing number of men to seek medical and surgical interventions aiming penile enlargement, even in the absence of anatomical abnormalities [4, 5]. 

Due to this rising demand, a spectrum of penile enhancement techniques has been described over the past decades. Minimally invasive approaches include injectable fillers such as hyaluronic acid (HA) and polylactic acid (PLA), which can offer temporary girth augmentation but are reabsorbed over time and, therefore, require repeated applications, increasing long-term costs and risks [6]. In parallel, the use of foreign substances through non-medical self-injection remains a concerning practice, often leading to severe complications [6]. 

In addition to injectable materials, several surgical strategies have also been described to augment penile dimensions, including autologous fat transfer, dermal matrices, subcutaneous silicone implants, and suspensory ligament release [7–10]. While many of these procedures have demonstrated promising short-term outcomes, they are frequently associated with postoperative complications such as infection, hematoma, fibrosis, and implant-related issues [6, 10]. Moreover, more invasive procedures, such as penile disassembly and total phalloplasty, can provide substantial gains but are complex and carry higher risks, including complications related to urethral anastomosis, flap viability, and multi-stage reconstruction [10]. 

Despite the variety of available techniques, current methods often fail to offer a definitive, durable, and biologically reliable outcome with an acceptable safety profile, underscoring the importance of advanced surgical approaches that provide more stable results while minimizing long-term complications [6, 10]. 

The dartos fascia flap has been described for penile girth augmentation [11]. Although this technique benefits from the use of autologous tissue, the dartos flap lacks an independent vascular pedicle, which may compromise its hydration and long-term volume maintenance, potentially resulting in outcomes that fall short of patient expectations [12]. In this context, a novel surgical technique named DART-VAG (Dartos and Tunica Vaginalis flap) is introduced, combining a dartos fascia flap with a tunica vaginalis flap to enhance both flap volume and vascularization. The addition of the tunica vaginalis aims to provide a more robust blood supply, potentially improving tissue viability, long-term stability, and aesthetic outcomes [12–14]. The present study reports the initial clinical experience with the DART-VAG technique, focusing on postoperative changes in penile length and girth, patient satisfaction, and its potential advantages over previously described penile augmentation methods.

## Materials and methods

### Study design

This was a prospective, non-randomized interventional study evaluating a novel penile girth augmentation technique, DART-VAG (Dartos and Tunica Vaginalis flap). Patients were consecutively enrolled between November 2021 and August 2025. There was no loss of patients during this period.

### Study population

Adult male patients undergoing penile girth augmentation using the DART-VAG technique during the study period were eligible for inclusion. All patients underwent preoperative consultation, during which they were counseled regarding expected surgical outcomes, postoperative care, recovery, and potential complications.

### Inclusion criteria

Men aged 18 years or older presenting with aesthetic dissatisfaction related to penile girth, with realistic expectations regarding surgical outcomes as assessed during preoperative consultation, were considered eligible for the study.

### Exclusion criteria

Patients with significant erectile dysfunction, Peyronie’s disease, active genital infection, decompensated psychiatric comorbidities, or suspected body dysmorphism based on preoperative clinical assessment and COPS-P screening were excluded.

### Surgical technique (DART-VAG)

**Fig. 1:**
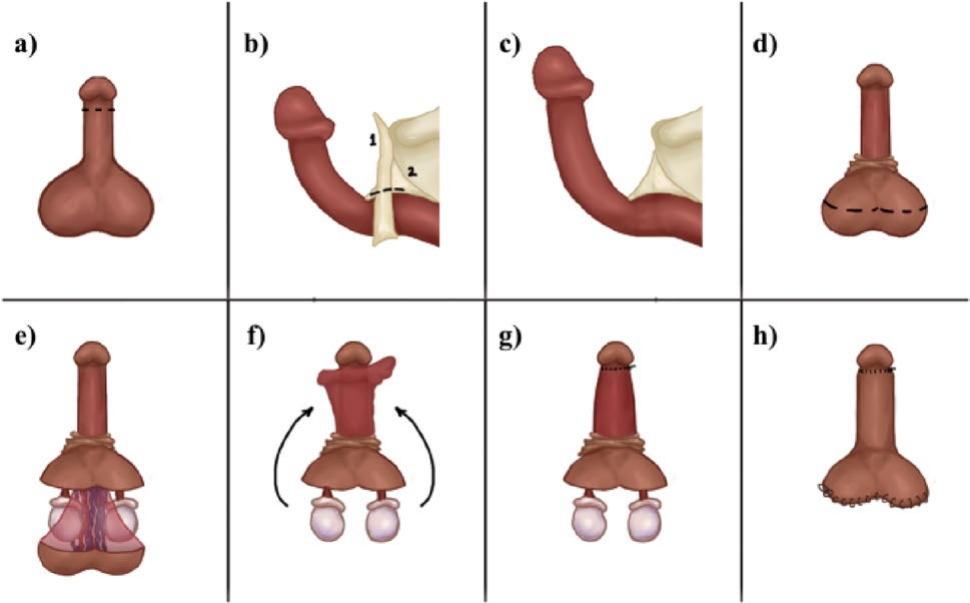
A schematic drawing is illustrated. **a** Penile incision. **b** Fundiform (1) and suspensory (2) ligaments are identified; only the fundiform is transected in its entire circumference. **c** View of the increased penile length after transection of the fundiform ligament. **d** Scrotal incision. **e** Flap of skin, dartos, and tunica vaginalis. **f** The flap is lifted to the penile shaft position. Skin is carefully removed, attempting to preserve the dermis. **g** The flap is sutured to the inner prepuce in all penile circumference. **h** Closure of the prepuce and scrotal incision

**Fig. 2:**
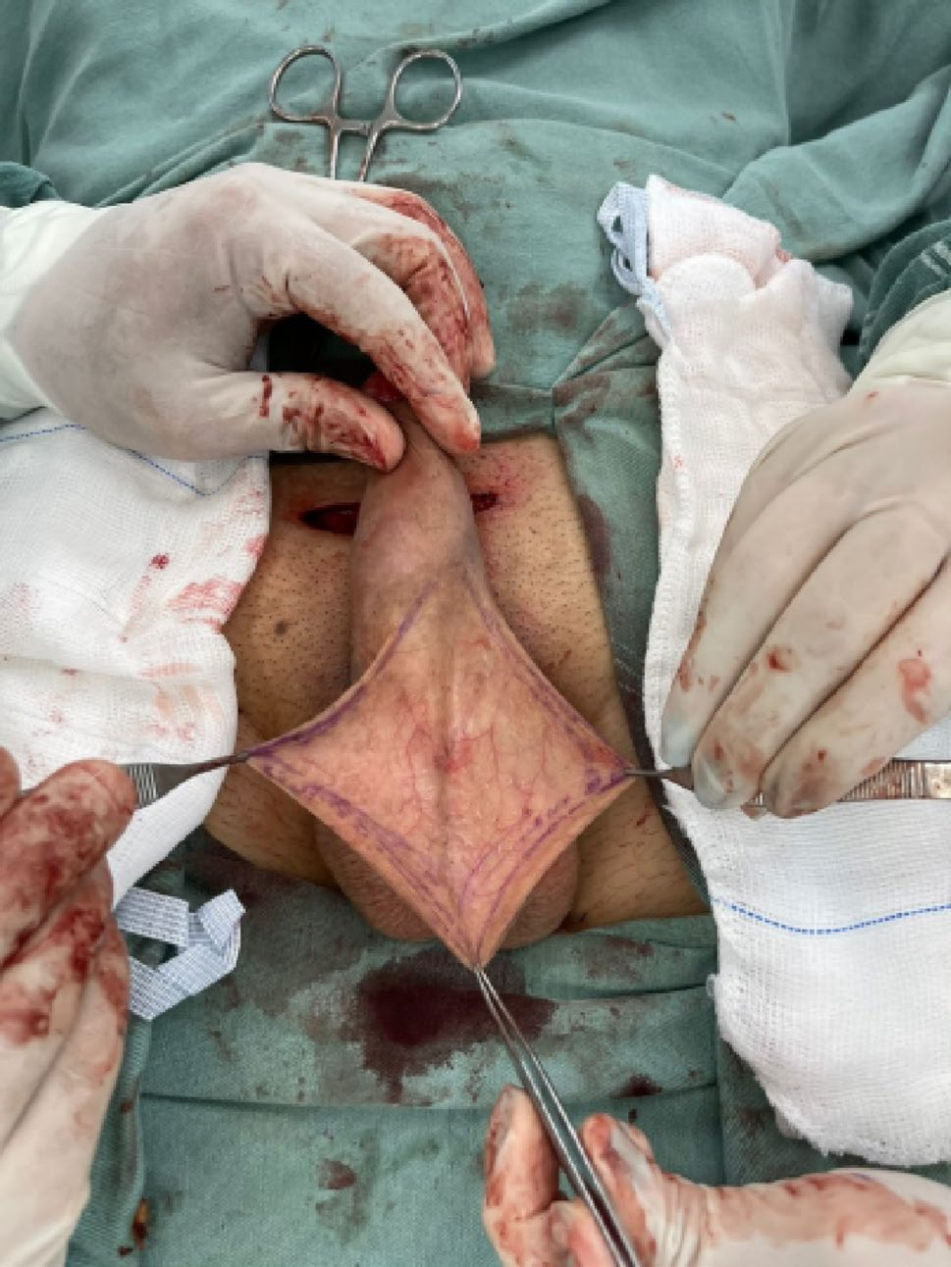
Diamond-shaped incision performed in cases of webbed penis

**Fig. 3:**
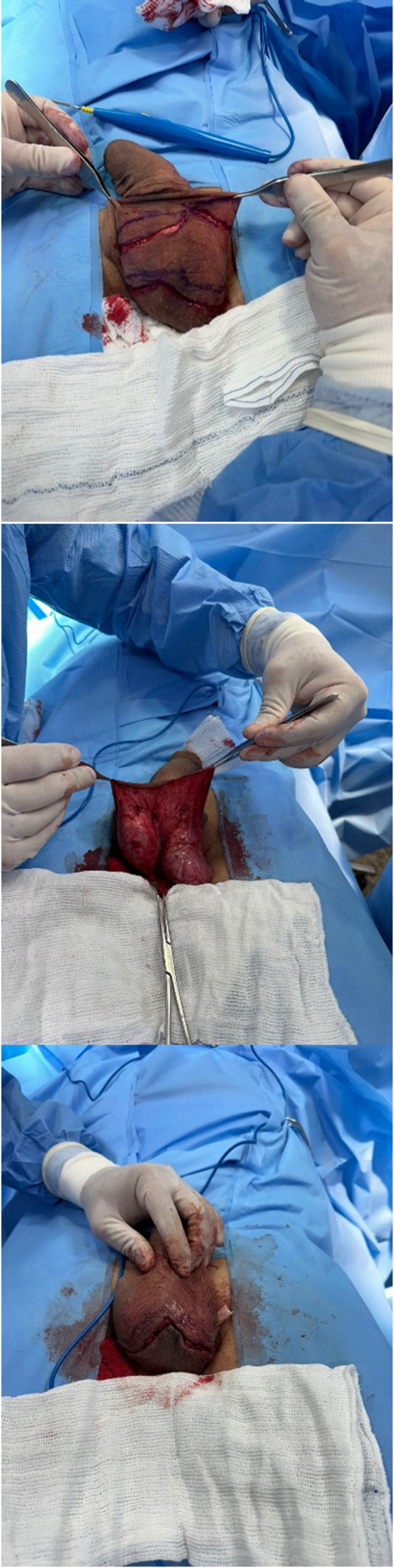
**a** Bat-shaped incision. **b** Dissection of the flap. **c** Appearance of the scrotal incision after penile shaft grafting

**Fig. 4:**
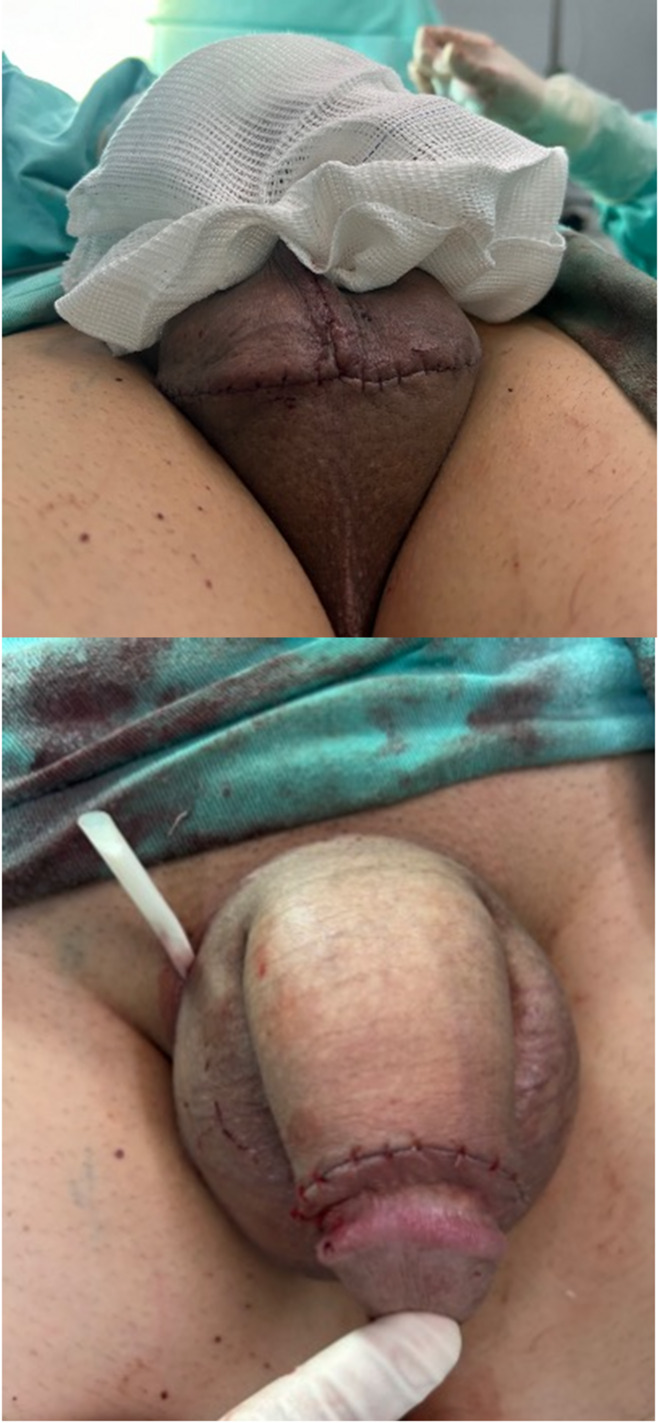
**a** Scrotal incision after wound closure. **b** Appearance of the penis immediately after surgery

**Fig. 5:**
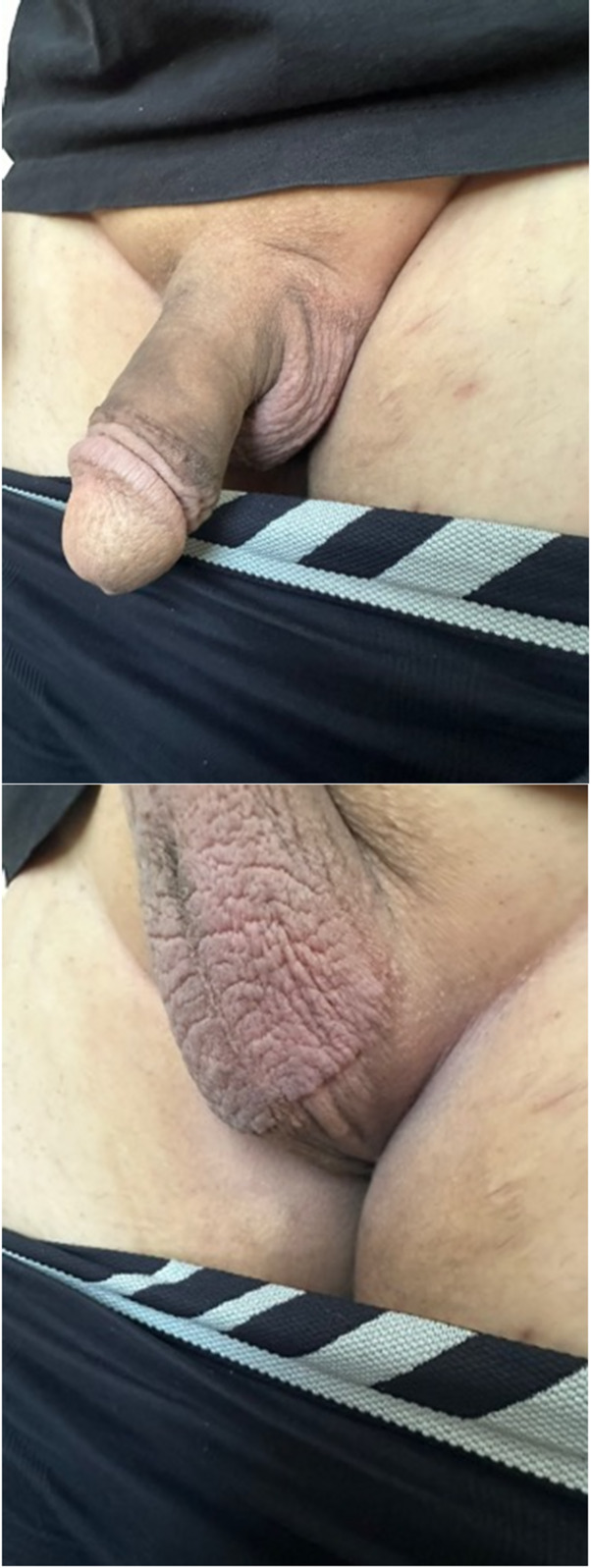
**a** Long-term follow-up of a patient who underwent the DART-VAG technique, showing a hidden scrotal incision. **b** View of the scrotal incision at the bottom of the scrotum, seen only when the patient lifts the scrotum

**Fig. 6:**
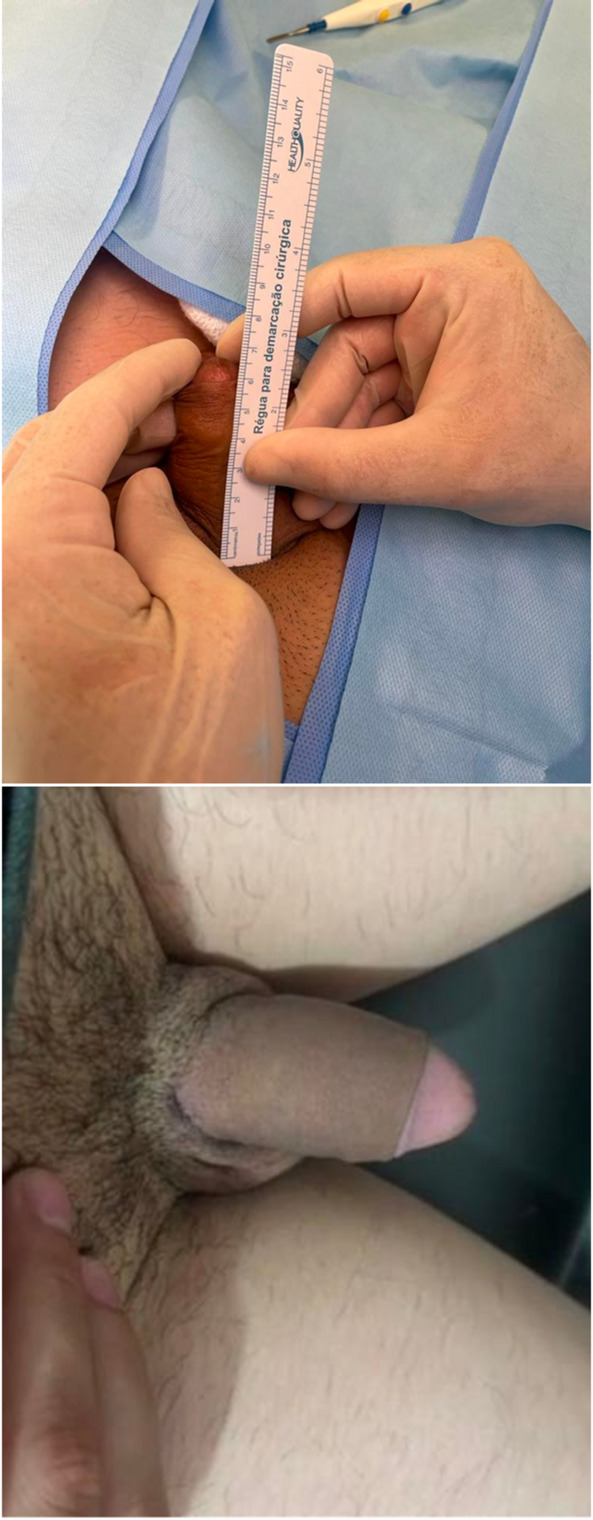
**a** Penis aspect before surgery. **b** Penis aspect after surgery

**Fig. 7:**
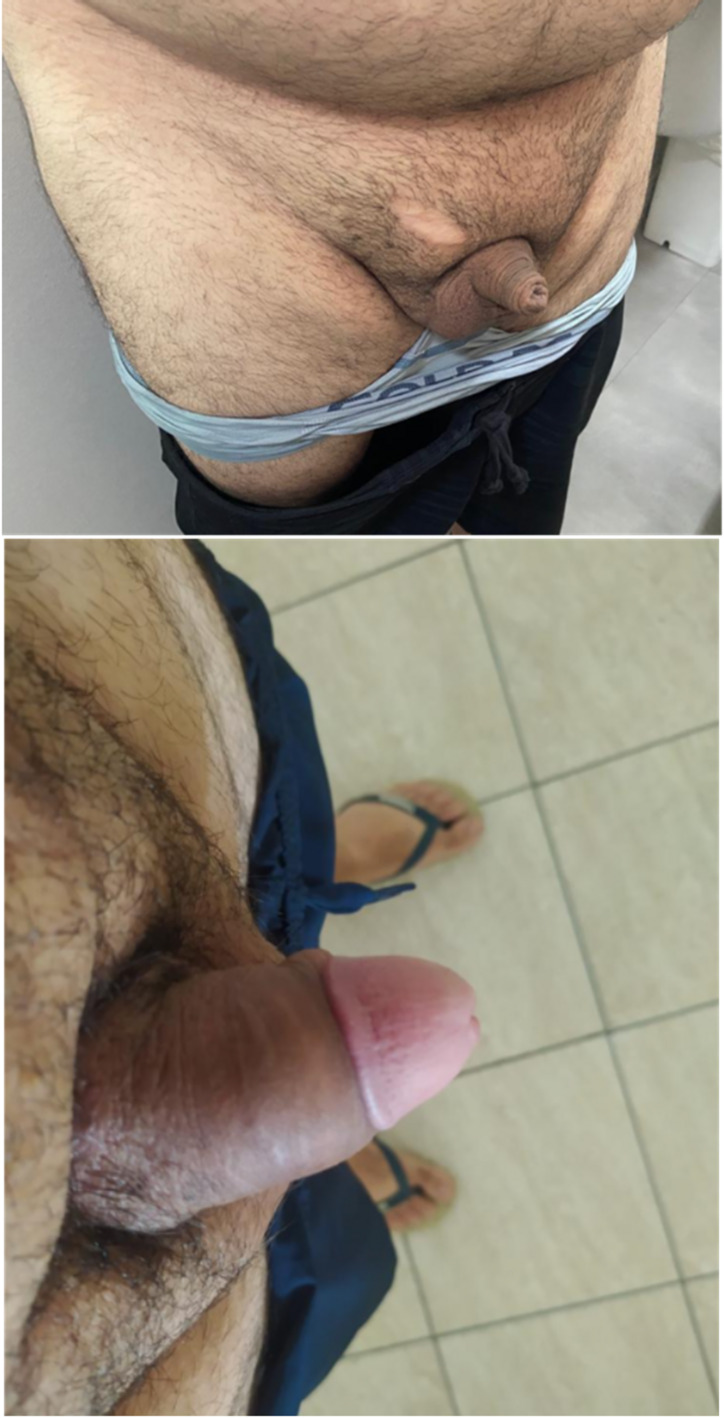
**a** Penis aspect before surgery. **b** Penis aspect after surgery

#### Anesthesia and patient positioning

The procedure was performed under spinal anesthesia, with the patient placed in the supine position. After hair removal and standard antiseptic preparation, sterile draping was applied.

#### Penile exposure

Mostly, a subcoronal circumferential incision was performed, followed by complete penile degloving. In the great majority of the patients, penile lengthening was needed. In this case, the fundiform ligament was identified and excised to facilitate penile mobilization, while the suspensory ligament was carefully preserved to maintain penile stability and support. In two patients who desired to preserve the prepuce, the penile shaft dissection was performed through a longitudinal penoscrotal incision along the scrotal raphe (Fig. 1).

#### Scrotal incision and flap harvesting

In cases where a webbed penis was present, the flap was harvested through a diamond-shaped incision made medially in the scrotum (Fig. 2). When no webbed penis was present, for aesthetic reasons, a bat-shaped incision at the bottom of the scrotum was performed. (Fig. 3) In this way, the scar is hidden, located at the lower edge or behind the scrotum (Figs. 4 and 5). Following exposure of the testicles, a composite flap consisting of dartos fascia and parietal tunica vaginalis was meticulously dissected. The tunica vaginalis was gently separated from the testis, preserving its vascular supply. The fat tissue surrounding the spermatic chord was also dissected and included in the flap. (Movie)

A subdartos tunnel was created at the penoscrotal junction for flap transposition. After flap harvesting, it was transposed to contact the penile shaft. Following transposition, complete de-epithelialization was performed, preserving the dermis as much as possible.

#### Flap transposition and fixation

The de-epithelialized dartos–tunica vaginalis composite flap was positioned circumferentially around the penile shaft. The flap was symmetrically wrapped to achieve uniform coverage and augmentation. Fixation was performed using absorbable sutures [e.g., 5 − 0 PDS], securing the flap in place and preventing displacement (Movie). The two lateral edges of the flap were sutured dorsally in the middle. Therefore, the entire penile shaft was covered by the flap.

#### Penopubic and penoscrotal fixation

To prevent penile length loss, penopubic and penoscrotal fixation with 3 − 0 PDS was performed at 12, 4, and 8 o’clock. For this fixation, the dermis was sutured to Buck’s fascia. Eventually, when the patient was circumcised, a VY plasty at the base of the dorsal side of the penis was performed to achieve a non-tense dorsal penile skin closure.

#### Closure and dressing

After meticulous hemostasis, scrotal skin closure was completed in layers. For a midline scrotal incision, closure was achieved using Z-plasty to reduce tension and optimize wound healing. A compressive dressing was applied. The placement of surgical drains was considered on a case-by-case basis.

#### Postoperative care

Patients were discharged the day after surgery. Standard postoperative recommendations included local wound care, activity restriction, and sexual abstinence for 8 weeks. Antibiotic prophylaxis was administered according to institutional protocol. To avoid penile fibrosis and loss of penile length, all patients were instructed to use weighted penile extenders (penile traction devices) for 30 min daily during the first postoperative month, until improvement of the healing process. Thereafter, conventional traction extenders were used for approximately 2 h per day, every day, for 6 months. These devices were self-administered by the patients following medical guidance. Figures 6 and 7 demonstrate long-term results.

### Evaluated variables and outcome measures

#### Penile morphometric assessment

Flaccid penile length was measured using a rigid ruler from the pubis to the tip of the glans, without compressing the pubic fat pad; therefore, the measurement corresponded to the apparent flaccid penile length. Accordingly, none of the patients met the criteria for micropenis. Penile circumference was measured using a flexible measuring tape at the mid-shaft region of the penis. Erect penile length was not measured, as no increase in erect length was expected; this was explained to all patients preoperatively.

Postoperative measurements were taken at the final follow-up visit, which occurred at least 6 months post-surgery. All preoperative and postoperative measurements were obtained by a single urologist, following a standardized protocol.

#### Psychosexual assessment

Psychosexual outcomes were assessed using two validated instruments: the Degree of Distress due to size concerns (DDC) questionnaire and the Male Genital Self-Image Scale (MGSIS).

The DDC was used to evaluate the level of psychological distress associated with concerns about penile size, with higher scores indicating greater distress. The MGSIS was employed to assess genital self-image, with higher scores reflecting a more positive perception of one’s genital appearance.

The DDC and MGSIS were administered preoperatively and at 6 months postoperatively. All instruments were assessed by one urologist through an in-person interview. Due to patient availability, only 10 patients could be assessed.

#### Patient satisfaction and self-perception

Patient satisfaction was assessed using a structured questionnaire based on a 5-point Likert scale (1 = very dissatisfied; 5 = very satisfied), administered during follow-up consultations. Patient self-perception and satisfaction with penile appearance were evaluated both before and after the procedure.

#### Postoperative follow-up

Postoperative follow-up visits were scheduled at predefined intervals to assess wound healing, complications, and patient-reported outcomes. The minimum follow-up duration considered for analysis was 6 months.

#### Complication assessment

Postoperative complications were prospectively recorded and classified according to the Clavien–Dindo system.

#### Statistical analysis

All statistical analyses were performed using SPSS software. Continuous variables were expressed as mean ± standard deviation. Pre- and postoperative comparisons were conducted using the paired t-test or the Wilcoxon signed-rank test, depending on data distribution. Statistical significance was set at *p* < 0.05.

## Results

A total of 32 patients underwent penile girth augmentation using the DART-VAG technique and were included in the analysis. The median age was 40.5 years (interquartile range [IQR]: 32–51 years).

### Surgical outcomes

The average penile length before surgery was 6.2 cm (*±* 1.67*)* and after surgery was 8.7 cm (*±* 1.46*)* (*p* < 0.001). The average penile girth before the procedure was 9.4 cm (*±* 1.08), and after increased to 11.2 cm (*±* 1.15) (*p* < 0.001). No patient reported loss of length or girth after the outcome measurement.

### Complications

No intraoperative complications were recorded. Four patients (12.5%) experienced wound dehiscence requiring specialized care. Among them, three (9.4%) healed by secondary intention with support from a specialized wound care nurse and were classified as Clavien–Dindo Grade I, whereas one (3.1%) required resuturing and was classified as Grade IIIb. One patient (3.1%) developed a scrotal hematoma requiring drainage in the operating room on the second day after surgery, which was also classified as Grade IIIb.

### Patient-reported outcomes

All patients (100%) were very satisfied or satisfied with the procedure and no one (0%) regretted it at the 6-month follow-up. After this period, a retrospective analysis of the patients’ data revealed that three patients (9.4%) requested hyaluronic acid injection for further girth enhancement. One patient (3.1%) who was initially satisfied with the procedure later expressed regret. He developed Peyronie’s disease two years after surgery and believed it was caused by the procedure. Figures 4 and 5 demonstrate long-term before and after results.

### Psychosexual outcomes

Preoperative assessment revealed psychological distress related to penile size, reflected in elevated DDC scores and reduced genital self-image as measured by the MGSIS.

Postoperative assessments revealed a decrease in overall distress (Table 1). Total DDC scores dropped significantly, with improvements observed across several individual domains, whereas a minority of items did not reach statistical significance.

Genital self-image also showed favorable postoperative changes (Table 2). Although the increase in the total MGSIS score did not reach statistical significance, several individual domains showed significant gains, suggesting improvement in specific aspects of genital self-perception.

Overall, these findings indicate a meaningful reduction in distress related to penile size concerns after surgery, together with domain-specific improvements in genital self-image. However, given the small number of patients who completed the psychosexual questionnaires, these results should be interpreted as preliminary and confirmed in larger studies with adequate statistical power.

**Table 1:** DDC before and after surgery

Variable	Before	After	*P* value
DDC total	40 (26.5–48.5)	23.5 (18.8–43.3)	0.01
DDC 1	0 (0–1.5)	4 (2–4)	0.02
DDC 2	8 (6.5–8)	4.5 (4–6)	0.01
DDC 3	2 (0–5)	5 (3.25–6)	0.17
DDC 4	6 (4.5–8)	4 (1.5–4.75)	0.01
DDC 5	6 (5–8)	4.5 (1.25–6)	0.03
DDC 6	2 (0–6)	0 (0–1.75)	0.14
DDC 7	2 (1–7)	2 (0–2.75)	0.06
DDC 8	8 (5–8)	2 (1.25–3.5)	0.01
DDC 9	6 (6–8)	1.5 (0–4)	0.02

Data are presented as median (interquartile range, IQR). *n* = 9 patients with paired preoperative and postoperative DDC data. Comparisons were performed using the Wilcoxon signed-rank test

**Table 2:** MGSIS before and after surgery

Variable	Before	After	*P* value
MGSIS total	12 (10–17)	16.5 (14–17.3)	0.09
MGSIS 1	1 (1–2)	2 (2–2)	0.04
MGSIS 2	1 (1–2)	2 (2–2)	0.02
MGSIS 3	2 (2–3)	3 (2–3)	0.04
MGSIS 4	1 (1–2)	2 (2–2)	0.01
MGSIS 5	3 (2–3)	3 (3–3)	0.094
MGSIS 6	3 (2–3)	2 (1–3)	0.73
MGSIS 7	1 (1–2)	2 (2–2)	0.01

Data are presented as median (interquartile range, IQR). *n* = 7 patients with paired preoperative and postoperative MGSIS data. Comparisons were performed using the Wilcoxon signed-rank test

## Discussion

In this study, we describe our experience with the DART-VAG technique to achieve circumferential penile enlargement. Thirty-two patients underwent the procedure, and measurable increases in both girth and length were observed. No intraoperative complications occurred, and there were no reports of loss of the achieved dimensions during follow-up, based on either in-office penile measurements or patient self-report. These findings support the conclusion that the technique is feasible and suggest that DART-VAG can reliably achieve volumetric enhancement, with positive early outcomes.

Changes were not confined to physical measurements. Distress related to penile size decreased substantially after surgery, as reflected by lower DDC total scores. The total MGSIS score did not reach statistical significance; however, improvements in several domains suggest a trend toward better genital self-image postoperatively. Particularly in men who seek intervention despite anatomically normal dimensions, validated patient-reported measures of distress and self-perception provide a necessary perspective on how structural change translates into psychological relief [15].

From a reconstructive standpoint, the rationale for integrating dartos tissue with a tunica vaginalis flap is to provide additional tissue volume while preserving vascular integrity and ensuring long-term stability. The tunica vaginalis is a delicate, mesothelium-lined membrane derived from peritoneal evagination that surrounds the testis anteriorly and laterally. Its flexibility and thinness allow it to adapt to nearby structures without adding excessive bulk. Because of its dependable blood supply and relative independence from scarred tissues, tunica vaginalis flaps are widely used in reconstructive urology [13, 14]. Since Snow BW first described its use to cover a reconstructed neourethra [16], it has been applied as a vascularized layer in various procedures, such as primary and redo hypospadias repairs and urethrocutaneous fistula reconstruction [17–20]. The consistent reporting of favorable healing outcomes in these settings reinforces its reliability as a vascularized adjunct in genital reconstruction, and supports its application in circumferential penile augmentation.

The increase in penile girth was statistically significant. Gains were greater in the first two postoperative months, likely due to local edema, then stabilized. Penile length also significantly increased postoperatively; however, some patients developed mild penile retraction secondary to penopubic fibrosis. In our experience, early intervention with dermatofunctional therapy and the use of penile extenders during the first six postoperative months helped prevent this loss. Although no studies have specifically evaluated this approach in penile lengthening, data from Peyronie’s disease suggest that traction therapy may also be beneficial in this context, in line with our clinical experience [21]. 

For penile length gain, we routinely section the fundiform ligament while preserving the main component of the suspensory ligament. The fundiform ligament is an anatomical landmark with no major supporting function [22, 23]. It arises from the anterior surface of the pubis, surrounds the base of the penis, and contributes to the formation of the septum between the two hemiscrotums. After circumferential release of the fundiform ligament, a significant increase in penile length can be achieved. We believe that sectioning the fundiform ligament provides substantial gain while avoiding erection instability risks linked to suspensory ligament release [24].

Another technical aspect of the DART-VAG technique is the scrotal incision design. When feasible, the incision is placed at the inferior aspect of the scrotum, allowing the final scar to remain concealed within the natural scrotal folds. This strategy reduces visible scarring in the resting position, which results in better aesthetic perception. Even in cases requiring alternative incision patterns, the closure is adjusted to minimize tension and optimize cosmetic outcomes. Since patients seeking penile augmentation are particularly sensitive to aesthetic details, scar concealment is key to their satisfaction.

Postoperative complications in this cohort were manageable. Wound dehiscence was the most frequent event and was treated conservatively in all cases except one, which required resuturing. A single patient developed a scrotal hematoma that was drained on postoperative day two. Flap necrosis and scrotal complications remain possible risks in procedures involving flap transposition and scrotal dissection, although flap necrosis was not observed in our series. Although the overall safety profile observed here is encouraging, penile augmentation procedures performed for cosmetic purposes call for careful patient counseling, as complication rates and dissatisfaction can be significant in the broader literature [6, 10, 25]. Expectations must therefore be addressed explicitly.

This study has limitations. It was not randomized, lacked a control group, and had a short follow-up period, which may limit the detection of late-onset complications and may overestimate patient satisfaction. Additionally, subjective outcomes were partially based on patient reports, which may be influenced by individual psychological factors. DDC and MGSIS assessments were only feasible in a subgroup of 10 patients operated on at one institution, of whom 9 and 7, respectively, agreed to complete the questionnaires. Although these patients were consecutively operated on, making major selection bias less likely, some degree of selection bias remains possible because only a subset completed these instruments. Larger prospective studies with longer follow-up and comparison groups are needed to determine durability, refine safety estimates, and clarify the precise role of DART-VAG within the spectrum of penile augmentation strategies.

## Conclusion

DART-VAG appears to be a feasible autologous technique for penile girth augmentation, providing significant increases in both penile length and girth at mid-term follow-up, with a manageable complication profile. Patient-reported outcomes improved after surgery, supporting its potential role in selected patients seeking durable girth enhancement. Larger studies with extended follow-up periods and comparative designs are necessary to confirm long-term durability and to better define indications.

## Electronic Supplementary Material

Below is the link to the electronic supplementary material.

Supplementary Material 1

## Data Availability

The datasets generated and/or analyzed during the current study are available from the corresponding author on reasonable request.
